# Management of von Willebrand Disease With a Factor VIII‐Poor von Willebrand Factor Concentrate: Results From the Paediatric Cohort of a Prospective Observational Post‐Marketing Study

**DOI:** 10.1111/hae.70129

**Published:** 2025-10-14

**Authors:** Jenny Goudemand, Annie Borel‐Derlon, Ségolène Claeyssens, Hervé Chambost, Guillaume Mourey, Annie Harroche, Sandrine Meunier, Céline Henriet, Thierry Leroi, Sophie Susen, Yohann Repesse

**Affiliations:** ^1^ Department of Haemostasis and Transfusion Lille University Hospital Lille France; ^2^ Haemophilia Treatment Centre University Hospital of Caen Caen France; ^3^ Haemophilia Treatment Center Purpan Hospital Toulouse France; ^4^ AP‐HM, Department of Paediatric Haematology Oncology Institut National De La Santé Et De La Recherche médicale, Institut national De La Recherche Agronomique Centre Recherche En CardioVasculaire et Nutrition Children Hospital La Timone & Aix Marseille University Marseille France; ^5^ Medical Biology Laboratory Biological Haemostasis Department CHU Besançon Besançon France; ^6^ CHU Besançon SINERGIES (UR 4662) Université Marie et Louis Pasteur Besançon France; ^7^ AP‐HP Haemophilia Treatment Centre Hospital Necker Paris France; ^8^ Hospices Civils de Lyon French Reference Center For Haemophilia Clinical Haemostasis Unit Lyon Louis Pradel Hospital Bron France; ^9^ Clinical Development Laboratoire Français Du Fractionnement et Des Biotechnologies (LFB) Les Ulis France; ^10^ Medical Operation France Laboratoire Français Du Fractionnement et Des Biotechnologies (LFB) Les Ulis France

**Keywords:** Factor VIII, paediatric, real‐life study, von Willebrand disease, von Willebrand factor, von Willebrand factor concentrate

## Abstract

**Introduction:**

Although clinical experience of a triple‐secured, plasma‐derived, von Willebrand factor (pdVWF), almost devoid of Factor VIII (FVIII) in adults with von Willebrand disease (VWD), is widely reported, its use in children is less documented.

**Aim:**

To explore the safety and efficacy of this concentrate in real‐life, in children <12 years old.

**Methods:**

Data from 30 paediatric patients enrolled in a prospective, 3‐year observational, post‐marketing study in France were analysed. Efficacy and safety were assessed in two cohorts: 0–6 and 6–11 years old.

**Results:**

The population included 14 children <6 years of age and 16 children aged 6–11 years. Most patients (80%) were severely affected (von Willebrand factor ristocetin cofactor activity [VWF:RCo] ≤ 15 IU/dL), including 30% with Type 3 VWD. Children received pdVWF for 16 major bleeds, 138 minor bleeds, 7 major surgeries, 8 minor surgeries and 12 invasive procedures. Efficacy was rated excellent or good in 100% of major bleeds or surgeries. The dose per infusion was approximately 50 IU/kg. By age group, median doses per infusion varied from 49 to 73 IU/kg for patients <6 years and from 46 to 59 IU/kg for patients 6–11 years, according to clinical situation. FVIII coadministration/correction was more frequent in Type 3 VWD, regardless of patient's age. Of five children receiving long‐term prophylaxis, breakthrough bleeding occurred in 1.6% of infusions and median annualised bleeding rate was 0.8. No safety concerns were raised.

**Conclusion:**

This analysis enlarges clinical experience of an FVIII‐poor pdVWF in the paediatric population. This concentrate offers safe and effective treatment regardless of VWD severity.

## 1 Introduction

Von Willebrand disease (VWD) is a common inherited bleeding disorder caused by quantitative (Type 1 and Type 3) or qualitative (Type 2) defects in von Willebrand factor (VWF), which stabilises Factor VIII (FVIII) and supports platelet adhesion [1, 2, 3]. Clinical presentation varies from mild mucocutaneous bleeding to severe musculoskeletal haemorrhage, depending on the VWF deficiency severity [3, 4].

The aim of treatment in children, as in adults, is to correct the primary VWF defect and secondary low FVIII levels. Except for certain forms of Type 2 and Type 3 VWD, both defects can be corrected by desmopressin [5]. However, desmopressin is contraindicated in children under 2 years old and should be used with caution in older children because of possible hyponatremia [6]. Thus, replacement therapy is of special importance in children with VWD. There are different VWF concentrates that contain variable proportions of FVIII [7]. A highly purified plasma‐derived VWF (pdVWF) almost devoid of FVIII, with a multimeric distribution close to that of VWF in normal plasma, has been routinely used in France since 1989 [8, 9]. The current formulation, WILFACTIN (LFB, France) referred as pdVWF in this paper, was approved in 2003 and recently received European approval for children aged <6 years, assisted by a paediatric study [10]. The present paper uses paediatric data collected during a previous post‐marketing study (PMS), which evaluated the long‐term safety and efficacy of pdVWF in a cohort of 158 patients, including 30 children aged <12 years, treated in real‐life medical practice [11].

## 2 Materials and Methods

### 2.1 Study Design

The study was a prospective, observational PMS conducted in 31 centres in France (2004–2009), which aimed to follow patients with inherited VWD for up to 3 years after enrolment [11]. Clinical situations in this paediatric cohort were analysed in two age groups: <6 years and 6–11 years.

### 2.2 pdVWF Used

WILFACTIN (or WILLFACT) is characterised by a high VWF‐specific activity and low FVIII content (residual FVIII:C ≤0.1 international units (IU) per 1 IU von Willebrand factor ristocetin cofactor activity [VWF:RCo]) [12]. It was used according to the Summary of Product Characteristics. If an immediate rise in FVIII:C was necessary, coadministration of a FVIII concentrate (plasma‐derived or recombinant) with the first infusion of VWF was recommended. For scheduled surgery, pdVWF treatment could be started 12–24 h before the procedure to allow sufficient increase of endogenous FVIII, with a second pdVWF dose administered just before the procedure (i.e., two preoperative infusions).

### 2.3 Clinical Assessment

Patients were evaluated at each clinic visit as previously described [11]. Data collection is presented in the Supporting Information. Efficacy by using a four‐point scale (excellent, good, moderate, and none) was only assessed at the last infusion for each major event as defined in Table S1. Long‐term prophylaxis (LTP) was evaluated by the number of breakthrough bleeding episodes that occurred within 3 days of the infusion. Annualised bleeding rate (ABR) was calculated from breakthrough bleeding events only for patients who received at least 12 months of treatment. Safety endpoints included monitoring of serious adverse events (SAEs) and both nonserious adverse events (AEs) and SAEs considered related to the product. Special attention was given to possible anaphylactic reactions, development of VWF inhibitors, modifications in viral status and thrombotic events. Descriptive statistics were performed using SAS version 9.1.3 (Cary, NC, USA).

## 3 Results

The demographic characteristics of the 30 patients <12 years old at study entry are shown in Table 1. Their ages ranged from 3 days to 11 years with a similar number of children in the two age groups. Sex ratio female/male was 1:1 in the <6 years group and 2:1 in 6–11 years group. Overall, 9 (30%) patients had Type 3 VWD, 18 (60%) had Type 2, 2 (6.6%) had Type 1 and 1 child had an unknown VWD type. Twenty‐four (80%) patients had basal VWF:RCo levels ≤15 IU/dL, which is defined as severe disease [13]. Twenty‐two (73.3%) patients had a basal level of FVIII:C ≤40 IU/dL, the minimum suitable haemostatic cutoff level [14]. None of the children had any history of inhibitors to VWF. Eighteen (60%), including three with Type 3 VWD, were considered as previously untreated patients (PUPs). Of these, 13 had never received any previous replacement VWF therapy, while five patients had been uniquely treated with pdVWF, started a few months before their inclusion.

**TABLE 1:** Patient demographics at inclusion by age group.

	Age groups		
	<6 years *N* = 14	6–11 years *N* = 16	All patients *N* = 30
Sex, female/male (*N*)	7/7	11/5	18/12
Age at inclusion (years)			
Median (range)	2 (3 days–5 years)	8 (6–11 years)	6 (3 days–11 years)
Weight at inclusion (kg)			
Median (range)	14.0 (3.5–22.5)	27.0 (19.1–45.6)	20.1 (3.5–45.6)
VWD type			
Type 1, *N* (%)	2 (14.3)	0 (0.0)	2 (6.6)
Type 2, *N* (%)	8 (57.1)^a^	10 (62.5)^b^	18 (60.0)^c^
Type 3, *N* (%)	3 (21.4)	6 (37.5)	9 (30.0)
Type unknown, *n* (%)	1 (7.1)	0 (0.0)	1 (3.3)
VWF:RCo plasma levels (IU/dL)			
Median (range)	13 (<1–36)	<5.0 (<1–17)	<6.0 (<1–36)
≤15 IU/dL, *N* (%)	9 (64.3)	15 (93.7)	24 (80.0)
VWF:Ag plasma levels (IU/dL)			
Median (range)	36 (1–72)	14 (<1–56)	18 (<1–72)
FVIII:C plasma levels (IU/dL)			
Median (range)	34 (1–81)	22.5 (<1–48)	23 (<1–81)
≤40 IU/dL, *N* (%)	7 (50.0)	15 (93.7)	22 (73.3)
<20 IU/dL, *N* (%)	5 (35.7)	7 (43.7)	12 (40)
Previously untreated patients (PUPs), *N* (%)^d^	12 (85.7)	6 (37.5)	18 (60)
Duration of follow‐up (months)			
Median (range)	26.4 (0.3–45.7)	27.9 (5.1–40.5)	27.9 (0.3–45.7)

Abbreviations: FVIII:C, Factor VIII coagulant activity; N, number of patients; VWD, von Willebrand disease; VWF:Ag, von Willebrand factor antigen; VWF:RCo, von Willebrand factor ristocetin cofactor activity.

^a^ Four patients had Type 2A VWD, one Type 2B and three unclassified Type 2 VWD.

^b^ Seven patients had Type 2A VWD, one Type 2B, one Type 2M and one unclassified Type 2 VWD.

^c^ Eleven patients had Type 2A VWD, two Type 2B, one Type 2M and four unclassified Type 2 VWD.

^d^ Previously untreated patients or patients treated only with Wilfactin.

All the 30 patients received at least one dose of pdVWF during the study and are included in the safety population. However, two received no treatment before their 12th birthday and are therefore excluded from the efficacy analyses.

### 3.1 Minor Bleeding Episodes

Seventeen patients experienced a total of 138 minor bleeds (Table 2). The median number of pdVWF infusions administered per episode was 1.7. The dose per infusion was slightly higher (55.0 IU/kg) for children <6 years old compared with those in the older group (47.2 IU/kg). A higher median total dose per episode was also observed in the younger group: 123.5 versus 86.9 IU/kg. Children with Type 3 VWD required a higher total dose per episode (125.7 IU/kg) than those with Type 2 (87.7 IU/kg). As expected, the frequency of coadministration of FVIII per episode was higher in patients with Type 3 VWD (86.8%) than those with Type 2 (15.7%). This difference was seen in both age groups.

**TABLE 2:** VWF treatment of minor bleeding episodes (*n* = 138).

	Age groups					
	<6 years		6–11 years		All patients	
Clinical setting	*N*	*n*	*N*	*n*	*N*	*n*
Number of patients (*N*); number of bleeding episodes (*n*)	7	36	12	102	17^a^	138
Type 1	0	0	0	0	0	0
Type 2	4^b^	13	8^c^	57	11^d^	70
Type 3	3	23	4	45	6	68
VWF treatment						
Number of infusions/episode						
Median (range)	1.6 (1.0–3.5)		1.8 (1.0–7.0)		1.7 (1.0–7.0)	
Type 2	1.8 (1.0–3.5)		1.8 (1.0–4.0)		1.7 (1.0–4.0)	
Type 3	1.6 (1.3–2.0)		2.2 (1.1–7.0)		1.8 (1.2–7.0)	
Number of exposure days/episode						
Median (range)	1.3 (1.0–2.6)		1.5 (1.0–7.0)		1.3 (1.0–7.0)	
Type 2	1.8 (1.0–2.6)		1.5 (1.0–4.0)		1.3 (1.0–4.0)	
Type 3	1.3 (1.3–2.0)		1.9 (1.0–7.0)		1.7 (1.0–7.0)	
VWF dose (IU/kg)/infusion						
Median (range)	55.0 (53.5–80.8)		47.2 (39.1–75.3)		50.0 (39.1–80.8)	
Type 2	54.4 (53.5–80.8)		47.2 (39.1–58.5)		50.0 (39.1–80.8)	
Type 3	64.0 (54.8–79.4)		46.5 (43.8–75.3)		56.5 (43.8–79.4)	
Total VWF dose (IU/kg)/episode						
Median (range)	123.5 (53.5–188.3)		86.9 (50.2–306.7)		98.8 (53.5–306.7)	
Type 2	112.7 (53.5–188.3)		86.9 (54.9–156.3)		87.7 (53.5–188.3)	
Type 3	123.5 (73.0–127.9)		144.1 (50.2–306.7)		125.7 (57.0–306.7)	
Coadministration of FVIII at first VWF infusion/episode, *N* (%)	26/36 (72.2)		44/102 (43.1)		70/138 (50.7)	
Type 2	4/13 (30.8)		7/57 (12.3)		11/70 (15.7)	
Type 3	22/23 (95.7)		37/45 (82.2)		59/68 (86.8)	

Abbreviations: FVIII, Factor VIII; VWF, von Willebrand factor.

^a^ Two patients are present in two age groups: <6 years and 6–11 years.

^b^ Two patients had Type 2A VWD, one Type 2B and one unclassified Type 2 VWD.

^c^ Five patients had Type 2A VWD, two Type 2B and one unclassified Type 2 VWD.

^d^ Seven patients had Type 2A VWD, two Type 2B and two unclassified Type 2 VWD.

### 3.2 Major Bleeding Episodes

Sixteen major bleeds were reported in nine children with Type 3 VWD (6), Type 2B (2) and unclassified Type 2 (1) (Table 3). Haemostatic efficacy was rated by investigators as ‘good’ or ‘excellent’ in all cases. Overall, the median number of infusions per episode was 6.5, and the median number of exposure days (EDs) was 5.5.

**TABLE 3:** VWF treatment of major bleeding episodes (*n* = 16).

	Age groups					
	<6 years		6–11 years		All patients	
Clinical setting	*N*	*n*	*N*	*n*	*N*	*n*
Number of patients (*N*); number of bleeding episodes (*n*)	4	4	5	12	9	16
Type 1	0	0	0	0	0	0
Type 2	1^a^	1	2^b^	9	3^c^	10
Type 3	3	3	3	3	6	6
VWF treatment						
Number of infusions/episode						
Median (range)	3.5 (2.0–7.0)		9.0 (4.0–24.0)		6.5 (2.0–24.0)	
Type 2	3.0 (3.0–3.0)		6.0 (4.0–19.0)		5.5 (3.0–19.0)	
Type 3	4.0 (2.0–7.0)		21.0 (13.0–24.0)		10.0 (2.0–24.0)	
Number of exposure days/episode						
Median (range)	3.5 (2.0–5.0)		8.0 (4.0–19.0)		5.5 (2.0–19.0)	
Type 2	3.0 (3.0–3.0)		6.0 (4.0–19.0)		5.0 (3.0–19.0)	
Type 3	4.0 (2.0–5.0)		16.0 (10.0–17.0)		7.5 (2.0–17.0)	
VWF dose (IU/kg)/infusion						
Median (range)	53.2 (51.3–77.7)		58.5 (45.8—95.2)		55.9 (45.8–95.2)	
Type 2	77.7 (77.7–77.7)		55.9 (45.8–95.2)		58.4 (45.8–95.2)	
Type 3	52.8 (51.3–53.6)		66.1 (56.0–76.0)		54.8 (51.3–76.0)	
Total VWF dose (IU/kg)/episode						
Median (range)	223.8 (105.6–359.1)		471.1 (217.5–1597.0)		388.0 (105.6–1597.0)	
Type 2	233.1 (233.1–233.1)		395.3 (217.5–1157.6)		388.1 (217.5–1157.6)	
Type 3	214.5 (105.6–359.1)		1344.1 (859.6–1597.0)		609.4 (105.6–1597.0)	
Coadministration of FVIII at first VWF infusion/episode, *N* (%)	3/4 (75)		4/12 (33.3)		7/16 (43.8)	
Type 2	0/1 (0)		1/9 (11.1)		1/10 (10)	
Type 3	3/3 (100)		3/3 (100)		6/6 (100)	
Efficacy ‘*Good*’ or ‘*Excellent*’, *N* (%)^d^	4/4 (100)		12/12 (100)		16/16 (100)	

*Note*: Treatment for major bleeding may include any further VWF received for the same event as an outpatient.

Abbreviations: FVIII, Factor VIII; VWF, von Willebrand factor.

^a^ Patient with unclassified Type 2 VWD.

^b^ Two patients with Type 2B VWD.

^c^ Two patients Type 2B VWD and 1 patient unclassified Type 2 WVD.

^d^ Efficacy considered ‘Good’ if bleeding stopped within the expected time period and ‘Excellent’ if bleeding stopped rapidly.

The older children received a higher number of infusions and for a longer period than the younger ones: median of 9 versus 3.5 infusions and 8 versus 3.5 EDs. However, the median dose per infusion was quite similar: 58.5 and 53.2 IU/kg, respectively. Bleeding episodes in patients with Type 3 VWD required a greater number of both EDs and infusions and consequently a higher total dose per episode than those with Type 2 VWD. However, the median dose per infusion was also quite similar for Type 2 (58.4 IU/kg) and Type 3 patients (54.8 IU/kg).

All patients experiencing major haemorrhage had basal VWF:RCo levels ≤15 IU/dL. The most frequent bleeding location was musculoskeletal in five (four Type 3 and one Type 2B) of the nine patients. One patient with Type 2B VWD suffered from repeated epistaxis. The others were related to intrabuccal events. A severe gingivorrhagia, in a patient with Type 2B VWD, required the use of two packs of red blood cells to overcome the drop in haemoglobin. FVIII was coadministered at the first infusion for 7 of the 16 major bleeds (43.8%), including all bleeds occurring in the six patients with Type 3 VWD and one bleed in a patient with Type 2B, despite his basal FVIII level >50 IU/dL. Management of each major bleed is detailed in Table S2.

### 3.3 Surgery

Fourteen patients were treated to prevent bleeding for 15 surgical procedures, 8 minor (Table 4) and 7 major (Table 5). For 9 of the 15 surgeries (60%), the FVIII level was increased either by FVIII administration with the first infusion of VWF or by two preoperative infusions of VWF. Correction of FVIII was more frequent in major surgeries (85.7% of cases) compared with minor surgeries (37.5%).

**TABLE 4:** VWF treatment for all (*n* = 15) and minor (*n* = 8) surgical procedures.

	Age groups					
	<6 years		6–11 years		All patients	
Clinical setting	*N*	*n*	*N*	*n*	*N*	*n*
**Overall surgical procedures**						
Number of patients (*N*), number of procedures (*n*)	7	7	7	8	14	15
VWF treatment						
Number of infusions/procedure						
Median (range)	8.0 (1–15)		5.5 (1–28)		6.0 (1–28)	
Number of exposure days/procedure						
Median (range)	7.0 (1‐10)		4.5 (1‐18)		5.0 (1‐18)	
VWF: dose (IU/kg)/infusion						
Median (range)	53.2 (37.3–83.1)		49.9 (37.4–57.0)		50.0 (37.3–83.1)	
Total VWF: dose (IU/kg)/procedure						
Median (range)	388.6 (83.1–650.7)		281.2 (39.6–1396.0)		283.7 (39.6–1396.0)	
Correction of FVIII, *N* (%)^a^	4/7 (57.1)		5/8 (62.5)		9/15 (60.0)	
**Minor surgical procedures** ^b^						
Number of patients (*N*), number of procedures (*n*)	3	3	4	5	7	8
VWF treatment						
Number of infusions/procedure						
Median (range)	2.0 (1–3)		2.0 (1–6)		2.0 (1–6)	
Number of exposure days/procedure						
Median (range)	1.0 (1–2)		2.0 (1–5)		2.0 (1–5)	
VWF dose (IU/kg)/infusion						
Median (range)	72.9 (53.2–83.1)		46.4 (37.4–56.7)		51.6 (37.4–83.1)	
Total VWF: dose/procedure (IU/kg)						
Median (range)	106.3 (83.1–218.7)		100.0 (39.6–283.7)		103.2 (39.6–283.7)	
Correction of FVIII, *N* (%)^a^	1/3 (33.3)		2/5 (40.0)		3/8 (37.5)	

Abbreviations: FVIII, Factor VIII; VWF, von Willebrand factor.

^a^ The FVIII level was increased either by a FVIII administration with the first infusion of VWF, or by two VWF preoperative infusions (additional infusion of VWF 12–24 h before the preprocedure dose).

^b^ Reparation of a cleft palate, laparotomy for exeresis of a testis residue and debridement of chalazia) in the <6‐year group and 4 tooth extractions and 1 repair of an inguinal hernia in the 6–11 year group.

**TABLE 5:** VWF treatment for seven major surgical procedures in seven patients.

Patient characteristics				Basal plasma levels at inclusion		Surgical procedures and treatment						
ID	Gender	Age (years)	VWD type	VWF:RCo (IU/dL)	FVIII:C (IU/dL)	Type of surgery	Number of infusions	Number of exposure days^a^	VWF per infusion (IU/kg)	Total VWF (IU/kg)	Correction of FVIII	Evaluation of efficacy^b^
**<6 years old**												
A	Male	3.5	2	14	59	Repair ectopic testis	9	7	72.3	650.7	2 preoperative VWF^c^	Good
B	Male	5.1	2A	28	81	Tonsillectomy	15	8	37.3	560.0	2 preoperative VWF^c^	Excellent
C	Female	5.5	2A	16	24	Tonsillectomy and adenoidectomy	13	10	49.5	643.5	2 preoperative VWF^c^	Good
D	Male	5.7	2	17	68	Circumcision due to phimosis	8	7	48.6	388.6	No	Excellent
						Median (range)	11 (8–15)	7.5 (7–10)	49.0 (37.3–72.3)	601.8 (388.6–650.7)		
**6–11 years old**												
E	Female	7.4	3	<6	<1	Tonsillectomy and adenoidectomy	28	18	49.9	1396.0	FVIII coadministration	Excellent
F	Female	9.0	2A	<5	37	Tooth extraction	17	12	51.7	878.3	FVIII coadministration	Good
G	Female	11.4	3	<6	1	Exeresis of angioma	11	9	57.0	627.3	FVIII coadministration	Excellent
						Median (range)	17 (11–28)	12 (9–18)	51.7 (49.9–57.0)	878.3 (672.3–1396.0)		
**Overall**												
						Median (range)	13 (8–28)	9 (7–18)	49.9 (37.3–72.3)	643.5 (388.6–1396.0)		

Abbreviations: FVIII, Factor VIII; FVIII:C, Factor VIII coagulant activity; VWD, von Willebrand disease; VWF, von Willebrand factor; VWF:RCo, von Willebrand factor ristocetin cofactor activity.

^a^ Total EDs may include any further treatment received for the same event as an outpatient.

^b^ Efficacy considered ‘Good’ if bleeding stopped within the expected time period and ‘Excellent’ if bleeding stopped rapidly.

^c^ Additional infusion of von Willebrand factor 12–24 h before the preprocedure dose.

### 3.4 Minor Surgical Procedures

Eight minor procedures were performed in seven patients: 3 in the <6 group and 5 in the 6–11 group (Table 4). The median number of infusions was 2.0 for both age groups, with two procedures requiring only a single infusion. The median VWF dose per infusion appeared to be higher in the <6‐year group (72.9 IU/kg) than in the older group (46.4 IU/kg). Nevertheless, the total dose received per procedure was similar, 106.3 and 100.0 IU/kg, respectively.

### 3.5 Major Surgical Procedures

Seven major procedures were performed in seven patients (Table 5). Efficacy was rated by investigators as excellent (*n* = 4) or good (*n* = 3) in 100% of the cases. All four younger children had Type 2 VWD and received a median dose per infusion of 49.0 IU/kg. The three older patients (two Type 3, one Type 2A VWD) required a similar median dose of 51.7 IU/kg. During the period covering hospitalisation and home treatment, the seven patients received a median number of 13 infusions for 9 EDs. Younger children received fewer infusions and were treated for a shorter time (median: 11 infusions and 7.5 EDs) than the older ones (median: 17 infusions and 12 EDs).

Correction of FVIII was implemented for all procedures in patients with Type 3 VWD and almost all in patients with Type 2 VWD, including two who had basal levels of FVIII:C of 59 and 81 IU/dL, respectively. There was no correction of FVIII for a circumcision in a patient with a basal FVIII level of 68 IU/dL.

### 3.6 Invasive Procedures

Five children were treated for 12 invasive procedures, one patient aged <6 years (Type 3 VWD) and four patients aged 6–11 years (three Type 3, one Type 2A). All procedures were treated by a single infusion at a median dose of 47.8 IU/kg (range 38.6–91.8). Patients with Type 3 VWD received coadministration of FVIII for six of seven procedures. The patient with Type 2A VWD did not receive any FVIII for five procedures.

### 3.7 Long‐Term Prophylaxis

Five patients were treated by LTP to prevent joint bleeding (three Type 3 patients), multiple bruising and traumatic bleeding of soft tissues (one Type 2B child) and epistaxis (one Type 3 child). The median age was 6.9 years (range 5.7–9.9). Only one patient (Type 3 VWD) was under 6 years old when LTP was initiated, and she subsequently continued LTP in the older age group (Table 6).

**TABLE 6:** Description of long‐term prophylaxis in five patients.

Patient characteristics at inclusion					Patient characteristics^a^	Long‐term prophylaxis and treatment					
ID	Gender	VWD type	VWF:RCo (IU/dL)	FVIII:C (IU/dL)	Age (years)	Prophylaxis period (months)	Number of infusions	Number of infusions per week	Dose per infusion (IU/kg)	Number of breakthrough bleeds^b^	Annualised bleeding rate^b^, ^c^
**Joint bleeding**											
H	Female	3	<4	1	5.7	33.5	17	1.6	50.3	0	0.0
					6.1		208	1.5	41.1	2	0.9
E	Female	3	<6	<1	7.4	31.4	263	2.3	76.2	7	2.7
G	Female	3	<6	1	9.9	28.7	240	1.9	51.1	0	0.0
					Median (range)	31.4 (28.7–33.5)		1.9 (1.5–2.3)	51.1 (41.8–76.2)		0.8 (0.0–2.7)
					Total		728			9/728 (1.2)	
**Other** ^d^											
I	Male	2B	11	54	6.5	15.2	72	ND	46.6	2	ND
J	Male	3	<3	1	6.9	1.1	2	ND	52.9	2	ND
					Median (range)	8.2 (1.1–15.2)		ND	49.8 (46.6–52.9)		ND
					Total (%)		74			4/74 (5.4)	
**Overall**											
					Median (range)	28.7 (1.1–33.5)		1.9 (1.5–2.3)	51.1 (41.8–76.2)		0.8 (0.0–2.7)
					Total (%)		802			13/802 (1.6)	

Abbreviations: FVIII:C, Factor VIII coagulant activity; ND, not determined; VWD, von Willebrand disease; VWF:RCo, von Willebrand factor ristocetin cofactor activity.

^a^ First infusion for LTP during the study.

^b^ Breakthrough bleeds included spontaneous or post‐traumatic bleeds which occurred within 3 days after infusion.

^c^ Annualised bleeding rate corresponds to the number of bleeding episodes per year in patients with at least one year of prophylaxis.

^d^ Other included multiple bruising and traumatic bleeding of soft tissues, and epistaxis.

The patients treated to prevent joint bleeding received a similar median dose per infusion (51.1 IU/kg), as those treated for other types of bleeding (49.8 IU/kg). Breakthrough bleeding occurred for 1.6% of infusions (13/802). Using the data from the three patients who underwent uninterrupted prophylaxis during an observation period of at least 1 year, the median ABR was 0.8.

### 3.8 Short‐Term Prophylaxis

Five patients (2 aged <6 and 3, 6–11 years old) with a basal VWF:RCo ≤ 15 IU/dL received a total of 11 prophylactic infusions (e.g., prevention of joint or muscle bleeding before physical activity), at a median dose of 52.7 IU/kg (range 34–78). No children experienced breakthrough bleeding within 3 days of infusion.

### 3.9 Safety

This real‐life study revealed no safety issues in the 30 children who received a total of 1731 infusions of pdVWF representing 3,016,073 IU of VWF. The median follow‐up was 27.9 months (range 0.3–45.7). No VWF inhibitors or allergic/anaphylactic‐type reactions were reported, particularly in the three Type 3 PUPs who totalised 13, 24 and 30 EDs, respectively.

Nine SAEs of moderate intensity and characteristic of a paediatric population were reported in four patients. All SAEs were judged unrelated to pdVWF and were resolved without sequelae (Table S3). None were thromboembolic events or modifications of viral status.

## 4 Discussion

Children have several characteristics that differentiate them from adults: frequent mucocutaneous bleeds [3], type of surgery, limited use of desmopressin [6] and early VWF exposure, with the risk of allergic reactions or inhibitors, particularly in Type 3 VWD. It is, therefore, of considerable interest to have details on replacement therapy in this population.

This prospective observational PMS assessed the safety and efficacy of a pdVWF in 30 children with VWD under 12 years old, including 14 (47%) under 6 years old. The majority (80%) presented with a severe form of the disease (VWF:RCo ≤15 IU/dL) including nine with Type 3 VWD.

### 4.1 Bleeding Episodes

Twenty children were treated for a total of 154 nonsurgical bleeding episodes. Most bleeds (90%) were minor, requiring generally fewer than two infusions of pdVWF at a dose of 50 IU/kg. Nine children with Type 2 or 3 VWD experienced 16 major bleeds treated at a median dose of 55.9 IU/kg and 6.5 infusions. Six were mucocutaneous, including one gastrointestinal haemorrhage and one severe gingivorrhagia, nine were musculoskeletal, including several knee or hip haemarthrosis and a psoas haematoma. These observations confirm the potential severity of some haemorrhagic events in a paediatric population and demonstrate that these events do not only occur in patients with Type 3 VWD. Despite the high severity of these episodes, all patients were successfully treated with pdVWF. The data are comparable with those observed in smaller series of bleeding events treated with double VWF/FVIII concentrates [15, 16, 17]. Most bleeds were minor. For major bleeds, the number of infusions was 2–8 in one study [15]; and 1–21 in another study [16]. In the third study, minor and major bleeds are not reported separately, 57.3% of bleeding episodes were treated by a single infusion, 26% by 2–8 and 16.7% did not require treatment [17].

### 4.2 Surgeries

The 15 surgical procedures described here were mostly typical of children, that is, orodental, ear–nose–throat (ENT) and circumcision. The dose per infusion was again approximately 50 IU/kg for minor or major surgeries and invasive procedures. Children received a single infusion for 14 out of 20 (70%) interventions, grouping minor and invasive procedures. As expected for major surgeries (7), the treatment duration was longer (median: 9 EDs, 13 infusions) to ensure complete wound healing. Efficacy was good or excellent in all cases. Our results, particularly for major surgeries, complement the few previously published cases treated with double concentrates [15, 16, 17].

### 4.3 LTP

Five children (4 with Type 3 VWD) received a total of 802 prophylactic infusions for a substantial median observation period of 28.7 months. In four cases, prophylaxis was initiated following a major bleeding episode described in this study. The treatment regimen consisted of approximately twice‐weekly infusions at a median dose of 51.1 IU/kg. Only 13 breakthrough bleeds occurred within 3 days of an infusion, that is, in 1.6% of infusions, testifying to the effectiveness of the LTP. This was reflected in the very low ABR 0.8 (range: 0–2.7).

Generally, LTP in children is poorly documented [15, 16, 17, 18, 19]. Evidence of bleeding reduction remains difficult to establish. In a study of 2:1 VWF/FVIII concentrate, the ABR in four children (3 with Type 3 VWD) <12 years old was 22.0 minor bleeds and 0.7 major bleeds, with a reduction of major bleeding only when compared to the previous year [17]. However, in a recent prospective study of 1:1 VWF/FVIII concentrate in nine children (5 with Type 3 VWD), aged 6–11 years, when 6 months of on‐demand treatment were compared to 12 months of LTP, the spontaneous ABR was reduced by approximately 95% with the disappearance of joint bleeding and a clear reduction in nose bleeds [19]. As in adults, LTP in children is initiated most frequently to reduce joint bleeding in patients with Type 3 VWD. However, LTP is also effective for mucosal bleeding, and not only in Type 3 VWD, as observed in our study.

### 4.4 FVIII Correction

Nearly 3/4 of the children treated had a baseline FVIII level of <40 IU/dL; therefore, coadministration of FVIII at the first VWF infusion would be expected since pdVWF is almost devoid of FVIII. This was globally the case in patients with Type 3 VWD (FVIII coadministration in 87% of minor bleeds and 100% of major bleeds and major surgeries). In patients with Type 2 VWD, it was rarer (FVIII correction in 16% of minor bleeds, 10% of major bleeds and 80% of major surgeries), generally because of sufficient basal FVIII levels. However, for 2 of 3 major surgeries (testicular repair and tonsillectomy) in children with basal FVIII levels >40 IU/dL, two pre‐operative doses of VWF were administered, probably as a precaution to ensure the best possible haemostatic conditions. Subsequent infusions were with pdVWF alone, demonstrating that only VWF is required after invoking a rapid increase in FVIII in the first hours of treatment, as previously reported [7, 11, 20].

### 4.5 Dosing

Clearly, the dose administered per episode for all clinical situations was higher in children with Type 3 VWD than those with Type 2 VWD, and thus comparisons between the two age groups depend on the proportion of patients/events of each type. However, a trend was observed towards a higher infusion dose in younger patients than in older patients, which may be explained by the study period, where pdVWF was only available in a 1000 IU VWF format. In addition, there is a general tendency, in the case of replacement therapies, to increase the posology in children compared with adults because of lower recovery in young children, classically attributed to an approximately 20% higher distribution volume [21]. Indeed, the mean recovery (SD) measured in seven children aged <6 years after a single infusion of pdVWF was 1.8 (0.4) IU/dL/IU/kg [10], while in eight adults it was 2.1 (0.3) IU/dL/IU/kg [22].

### 4.6 Importance of PUPs

From our population considered as PUPs (18 including three Type 3 VWD), one can see that no allergic reactions or anti‐VWF antibodies were observed, as was the case in Gouider et al., where the three PUPs (all Type 3 VWD) were followed for at least 18 months [10]. The timing of antibody development is uncertain, but likely to coincide with first exposures. Only two reports with other concentrates describe this type of adverse reaction: a rash in a 4‐year‐old child (Type 3 VWD) treated by LTP with a 1:1 VWF/FVIII concentrate [16] and the development of VWF inhibitors in a 7‐year‐old girl (Type 3 VWD) during intensive treatment with a 2:1 VWF/FVIII concentrate [23].

### 4.7 Strengths and Limitations

This study has several strengths: it is a prospective, multicentre study involving a group of 30 children, nearly half of whom were very young (<6 years), treated in various clinical settings typical of paediatric practice, with systematic and detailed data collection over a period of more than 2 years.

However, it also has limitations, primarily related to the young age of this population, which restricted the sample size and, consequently, the number of episodes analysed particularly the most severe ones. Additionally, this was an observational study aimed at providing Supporting Information data to already established findings.

## 5 Conclusion

Together with the study by Gouider et al. [10], the data collected here confirm the efficacy of Wilfactin in children under 12 years of age with various severities of VWD. This study provides reassurance for the use of a pdVWF almost devoid of FVIII in paediatric populations. The treatment regimens, which consider basal FVIII levels, are comparable with those used in adults. No safety issues occurred during the long observation period of up to 3 years.

## Author Contributions

J.G. was a study investigator and supervised and wrote the manuscript. C.H. was involved in the concept and design of the study and the analysis and interpretation of data, and wrote the manuscript. T.L. supervised the manuscript and contributed to the interpretation of data. S.C., H.C., A.H., S.M., G.M., S.S. and Y.R. were primary investigators for the study or supervised the manuscript. A.B.‐D. was a study investigator and contributed to the design of the study and interpretation of data. All authors reviewed and approved the final manuscript.

## Ethics Statement

The study was a noninterventional study exempted from the requirements for an independent Ethics Committee review. The study was conducted in France in accordance with Ethical principles derived from the Declaration of Helsinki and the Good Clinical Practices (CPMP/ICH/135/95) and the French Public Health Code as modified by the law dated 18 January 1994 (article L.365.1). In accordance with French law adopted on 6 January 1978 concerning information technology and personal privacy and the law adopted on 1 July 1994 concerning the use of nominative data for health research, the protocol and case report form were submitted for approval to the Advisory Committee for the Treatment of Data for Health Research (Comité Consultatif sur le Traitement de l'Information en Matière de Recherche dans le Domaine de la Santé) and declared to the French Data Protection Committee (Commission Nationale de l'Informatique et des Libertés).

## Conflicts of Interest

This work was funded by LFB, Les Ulis, France. Jenny Goudemand received fees and funding from LFB and Roche. Annie Borel‐Derlon received fees for participation on the advisory board for LFB and for oral presentation for Sobi. Ségolène Claeyssens, Guillaume Mourey and Sandrine Meunier have no conflicts of interest to declare. Hervé Chambost received fees and funding from CSL Behring, LFB, Roche Chugaï, Sobi, Novo Nordisk and Octapharma (speaker at symposium, congress, consulting and expert boards). Annie Harroche received fees and funding from LFB, CSL Behring, Roche, Sobi, Novo Nordisk, Takeda, Octapharma (speaker at symposium, congress, consulting and expert boards). Sophie Susen received research support/P.I. from Biomarin, Bioverativ, CSL Behring, CorWave, Roche‐Chugai, Sanofi, Shire/Takeda, Siemens Healthineers, Sobi, and Stago LFB and fees for Scientific Advisory Board from Biomarin, CSL Behring, LFB, Roche, Novo Nordisk, Sanofi, Sobi and Takeda. Yohann Repesse received funding (fees for speaking and consulting) from LFB. Céline Henriet and Thierry Leroi are employees of LFB.

## Supporting information




**Supporting Table 1**: Definitions of severity of events and haemostatic response to treatment.
**Supporting Table 2**: VWF treatment for 16 major bleeding episodes in 9 patients.
**Supporting Table 3**: Overview of reported adverse events by age group (age at inclusion).

## Data Availability

The data that support the findings of this study are available from the corresponding author upon reasonable request.
