# Supplementary material for: Management of von Willebrand Disease With a Factor VIII‐Poor von Willebrand Factor Concentrate: Results From the Paediatric Cohort of a Prospective Observational Post‐Marketing Study
Source: Haemophilia. 2025 Oct 14;31(6):1250–60. doi: 10.1111/hae.70129 (PMC12612368; doi:10.1111/hae.70129)
Supplement: Supplementary file 1 — Supporting Table 1: Definitions of severity of events and haemostatic response to treatment. Supporting Table 2: VWF treatment for 16 major bleeding episodes in 9 patients. Supporting Table 3: Overview of reported adverse events by age group (age at inclusion). [file HAE-31-1250-s001.docx]

**“*Management of von Willebrand disease with a factor VIII-poor von Willebrand factor concentrate: Results from the paediatric cohort of a prospective observational post-marketing study.”***

**SUPPORTING INFORMATION**

**MATERIALS AND METHODS**

*Data collection*

Two children <6 years old moved to the 6-11 year group during the study, and were therefore analysed in both age groups.

The number of VWF infusions, exposure days (EDs), units infused (VWF:RCo), co-administration of FVIII or the use of two preoperative infusions, and administration of red blood cells were recorded at each clinical visit.

Major bleeds and major surgeries were defined according to the number of EDs and/or need for hospitalisation (Table S1). Efficacy was assessed for major events only.

For long-term prophylaxis (LTP), the number of breakthrough bleeding episodes that occurred within 3 days of the infusion was collected. The prophylaxis duration was defined as the period between the first and the last visit recording LTP infusions. As exact dates of start and end of LTP were not recorded, the frequency of infusions was determined only for periods of definite LTP.

Short-term prophylaxis was defined by administrations over relatively brief periods of time to prevent bleedings commonly known to occur in daily life (e.g. prior to physical activity).

Safety endpoints included monitoring of serious adverse events (SAEs), as defined in the Good Clinical Practice guidelines of EU directive 2001/20/EC, and both non-serious adverse events (AEs) and SAEs considered related to the product.

**Table S1** Definitions of severity of events and haemostatic response to treatment

|  | Bleeding episodes | Surgical procedures | Invasive procedures |
| --- | --- | --- | --- |
| Classification of events | | | Dental care (including dental scaling), ear-nose-and throat interventions, intra-articular injection and other non-surgical/diagnostic procedures without biopsy such as endoscopy |
| *Major* | Requiring hospitalisation and >3 days of treatment (or less according to the investigator's discretion) | Any procedure covered by >5 days of treatment |  |
| *Minor* | Bleeding treated on an out-patient basis or ≤3 days at hospital | All procedures covered by ≤5 days of treatment |  |
| Rating of haemostatic response by the investigator (**major events only**) | | |  |
| *Excellent* | Bleeding stopped rapidly | Clinically normal haemostasis |  |
| *Good* | Bleeding stopped within the expected time period | Light bleeding at surgical incision | Haemostatic efficacy was not recorded for invasive procedures in the study |
| *Moderate* | Bleeding controlled with difficulty | Moderate, but controlled bleeding |  |
| *None* | Uncontrolled bleeding | Severe, uncontrolled bleeding |  |

**Table S2** VWF treatment for 16 major bleeding episodes in 9 patients

| Patient characteristics  at inclusion | | | | | Patient characteristics  at visit | | Major bleeding episodes and treatment | | | | | | | |
| --- | --- | --- | --- | --- | --- | --- | --- | --- | --- | --- | --- | --- | --- | --- |
| **ID** | **Gender** | **VWD type** | **VWF:RCo (IU/dL)** | **FVIII:C**  **(IU/dL)** | **Age (years)** | **Weight (kg)** | **Location** | **Reported terms** | **Number of infusions** | **Number of exposure days^†^** | **Dose per infusion (IU/kg)** | **Total dose (IU/kg)** | **Co-ad FVIII at 1^st^ infusion** | **Evaluation of efficacy** |
| <6 years old | | | | | | | | | | | | | | |
| K | Male | 3 | <2 | 3 | 1.4 | 9.8 | Gastro-  intestinal | Rectorrhagia associated with intestinal invagination | 2 | 2 | 52.8 | 105.6 | Yes | Good |
| L | Female | 3 | <12 | 1 | 2.0 | 11 | Intra buccal | Tongue bite | 7 | 5 | 51.3 | 359.1 | Yes | Good |
| M^‡^ | Male | 2 | 14 | 44 | 2.1 | 13 | Other | 14h after cryptorchidism + hernia repair | 3 | 3 | 77.7 | 233.1 | No | Good |
| H | Female | 3 | <4 | 1 | 5.7 | 20 | Musculo  skeletal | Haemarthrosis right knee | 4 | 4 | 53.6 | 214.5 | Yes | Good |
| 6-11 years old | | | | | | | | | | | | | | |
| I | Male | 2B | 11 | 54 | 6.5 | 25 | ENT | Epistaxis | 4 | 4 | 76.5 | 306.0 | Yes | Good |
|  |  |  |  |  |  |  | ENT | Epistaxis | 4 | 4 | 95.2 | 380.8 | No | Good |
|  |  |  |  |  |  |  | ENT | Epistaxis + haematomas | 5 | 4 | 45.8 | 228.8 | No | Good |
| E | Female | 3 | <6 | <1 | 7.4 | 20.1 | Musculo  skeletal | Bilateral hip haemarthrosis | 21 | 16 | 76.0 | 1 597.0 | Yes | Good |
| N | Female | 2B | <5 | 33 | 8.4 | 40.0 | Musculo  skeletal | Haematomas after falling from bicycle | 8 | 8 | 55.9 | 447.0 | No | Good |
|  |  |  |  |  |  |  | Musculo  skeletal | Cervical haematoma | 4 | 4 | 54.4 | 217.5 | No | Good |
|  |  |  |  |  |  |  | Musculo  skeletal | Haematoma on thigh after falling from bicycle | 10 | 8 | 50.8 | 508.3 | No | Good |
|  |  |  |  |  |  |  | Musculo  skeletal | Haematoma right thigh | 10 | 10 | 49.5 | 495.3 | No | Good |
|  |  |  |  |  | 9.5 | 48.8 | Intra  buccal | Gingivorrhagia**^§^** | 19 | 19 | 60.9 | 1157.6 | No | Good |
|  |  |  |  |  |  |  | Musculo  skeletal | Haematoma on back | 6 | 6 | 65.9 | 395.3 | No | Excellent |
| G | Female | 3 | <6 | 1 | 9.4 | 27.5 | Musculo  skeletal | Haemarthrosis right hip | 13 | 10 | 66.1 | 859.6 | Yes | Good |
| O | Male | 3 | <3 | <1 | 11.9 | 41.0 | Musculo  skeletal | Haematoma psoas | 24 | 17 | 56.0 | 1344.1 | Yes | Good |

Abbreviations: ENT: Ear, Nose & Throat; FVIII:C, factor VIII coagulant activity; VWD, von Willebrand disease; VWF:RCo, von Willebrand factor ristocetin cofactor activity.

^†^Total EDs may include any further treatment received for the same event as an outpatient; ^‡^Patient entered study after intervention had been carried out; ^§^Patient was hospitalised due to serious gingivorrhagia, which was treated with 3000 IU of pdVWF 3x a week and 2 packs of red blood cells when hospitalised.

Table S3 Overview of reported adverse events by age group (age at inclusion)

| Adverse events (AEs) | | **<6 years** | **6-11 years** | **All patients** |
| --- | --- | --- | --- | --- |
| Number of patients (number of infusions) | | 14 (498) | 16 (1233) | 30 (1731) |
| Number of infusions by patient: median (range) | | 9.0 (1-253) | 15.0 (2-369) | 10.5 (1-369) |
| Duration of follow-up in months: median (range) | | 26.4 (0.3-45.7) | 27.9 (5.1-40.5) | 27.9 (0.3-45.7) |
| AE of special interest  Presence of VWF inhibitors  Allergic reactions  Transmission of infectious agents  Thromboembolic events | | 0  0  0  0 | 0  0  0  0 | 0  0  0  0 |
| Serious AEs^†^ | | *N (%) E* | *N (%) E* | *N (%) E* |
| All  Intensity  Very severe  Severe  Moderate  Mild | | 2 (14.3) 6  0  0  2 (14.3) 6  0 | 2 (12.5) 3  0  0  2 (12.5) 3  0 | 4 (13.3) 9  0  0  4 (13.3) 9  0 |
| Seriousness criteria | |  |  |  |
| Requires or prolongs hospitalization | | 2 (14.3) 6 | 2 (12.5) 3 | 4 (13.3) 9 |
| Relationship to the investigational product | |  |  |  |
| Not related |  | 2 (14.3) 6 | 2 (12.5) 3 | 4 (13.3) 9 |
| Related |  | 0 | 0 | 0 |

Abbreviations: N, number of patients; E, number of adverse events.
^†^All SAEs were moderate, defined as an event that requires or prolongs hospitalisation. Adverse events included 5 falls resulting in head or face injury, 1 case of intussusception, 1 parasitic infection, 1 urinary tract infection and 1 case of menometrorrhagia. All were satisfactorily resolved without sequelae.
